# The Inflammation‐Initiating and Resolving Mechanisms and Oxidation: Could Periodontal Therapy and Nutritional Strategy Improve the Systemic Health? A Narrative Review

**DOI:** 10.1002/fsn3.70096

**Published:** 2025-03-20

**Authors:** Roberta Salvatori, Luigi Generali, Elisa Bellei, Stefania Bergamini, Carlo Bertoldi

**Affiliations:** ^1^ Department of Medical and Surgical Sciences for Children and Adults University of Modena and Reggio Emilia School of Medicine Modena Italy; ^2^ Department, of Surgery, Medicine, Dentistry and Morphological Sciences With Transplant Surgery, Oncology and Regenerative Medicine Relevance University of Modena and Reggio Emilia Modena Italy

**Keywords:** cardiovascular diseases, diabetes, inflammation, periodontitis, rheumatoid arthritis

## Abstract

Periodontitis (PDS) is one of the most common and crippling systemic diseases. It is a chronic inflammatory condition that leads to the loss of periodontal attachment, resulting in tooth loss. In addition to its effects on oral health and nutrition, PDS is closely linked to other systemic conditions such as diabetes mellitus (DM), cardiovascular diseases (CVD), and rheumatoid arthritis (RA). The active role of inflammation and oxidation in systemic health, as well as their relationship with periodontal therapy, has been investigated. This review explores the evidence on how periodontal therapy and dietary lifestyle can help reduce chronic inflammation, limit oxidation, and prevent related pathologies. Nonsurgical periodontal therapy (NSPT) and nutrition have been extensively discussed as potential contributors to positive clinical outcomes by resolving inflammatory pathogenic pathways. NSPT, foods, and dietary supplements represent therapeutic strategies that address the underlying mechanisms of chronic inflammation and oxidation at various stages. A key finding from this review is that periodontal treatment, in conjunction with nutritional counseling, can improve clinical outcomes in PDS, DM, CVD, and RA. However, for NSPT, nutrition, and dietary composition to be effective, they should be integrated into a sustainable, long‐term lifestyle.

## Graphical abstract

This review examines the role of nonsurgical periodontal therapy (NSPT) and nutrition in managing periodontitis and associated systemic diseases, such as diabetes, cardiovascular diseases, and rheumatoid arthritis. Findings suggest that combining NSPT with dietary interventions can help reduce inflammation and oxidative stress, but long‐term success requires integrating these strategies into a sustainable lifestyle.

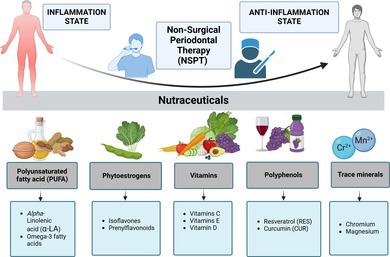

**Abbreviations**
AA: arachidonic acid
ap: apigenin
COX: cyclooxygenase
CURs: curcumins
CVD: cardiovascular diseases
CVS: cardiovascular system
DALYs: disability‐adjusted life years
DHA: docosahexaenoic acid
DM: diabetes mellitus
DPA: docosapentaenoic acid
EGCG: epigallocatechin gallate
EPA: eicosapentaenoic acid
HbA1c: hemoglobin A1c
LOX: lipoxygenase
LT5: series‐5 leukotrienes
MeSH: medical subject headings
MMPs: matrix metalloproteinases
NHANES: National Health and Nutrition Examination Survey
NSPT: nonsurgical periodontal therapy
PDS: periodontitis
PG3: series‐3 prostaglandins
PMNs: polymorphonuclear neutrophils
PUFAs: polyunsaturated fatty acids
QUE: quercetin
RA: rheumatoid arthritis
RES: resveratrol
ROS: reactive oxygen species
Rv: resolvin
SPMs: specialized proresolving molecules

## 1 Introduction

The oral cavity is characterized by an important and peculiar type of joint, the periodontium, which stabilizes the teeth to the alveolar bone, allowing proper masticatory function and nutrition (Bertoldi et al. 2024) (Sanz et al. 2020a). Periodontitis (PDS) is a chronic inflammatory disease, triggered by oral microbiota, and it is one of the most frequent diseases worldwide (Bertoldi et al. 2024) (Kassebaum et al. 2014).

Inflammation is an evolutionarily conserved process that protects the host from noxious agents, such as bacteria, viruses, toxins, and infections by removing pathogens and inducing tissue repair and healing (Netea et al. 2017).

Among the most important clinical discoveries of the last few decades are the involvement of the immune system and inflammatory processes in a large number of health problems that dominate prevailing morbidity and mortality worldwide (Slavich 2015). Moreover, chronic inflammatory diseases represent the most significant cause of more than 50% of all deaths worldwide (*Lancet (London, England)* 2018). The risk of developing chronic inflammation should be traced back to early development (Fleming et al. 2018). The effects of chronic inflammation are now known to continue throughout the life span to impact adulthood health and disability or even risk of mortality (Fleming et al. 2018).

**FIGURE 1:**
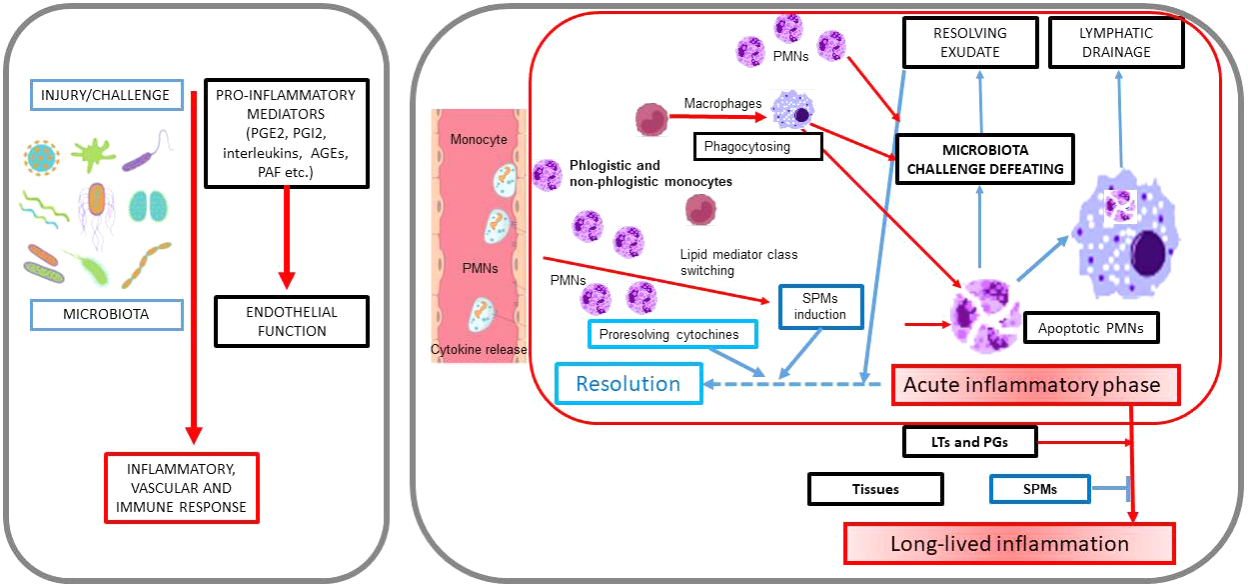
The acute inflammatory pathway and its possible resolution. Legend: Red arrows indicate a prevailing proinflammatory activity, whereas blue arrows show a prevailing proresolving activity. Red borders describe inflammatory phases, blue borders indicate proresolving phases and black borders symbolize the environment in which the different phases of inflammation occur. AGEs, advanced glycation end‐product; LTs, leukotrienes; PAF, platelet‐activating factor; PG, prostaglandins; PMNs, polymorphonuclear neutrophils; and PMs, specialized proresolving mediators.

**FIGURE 2:**
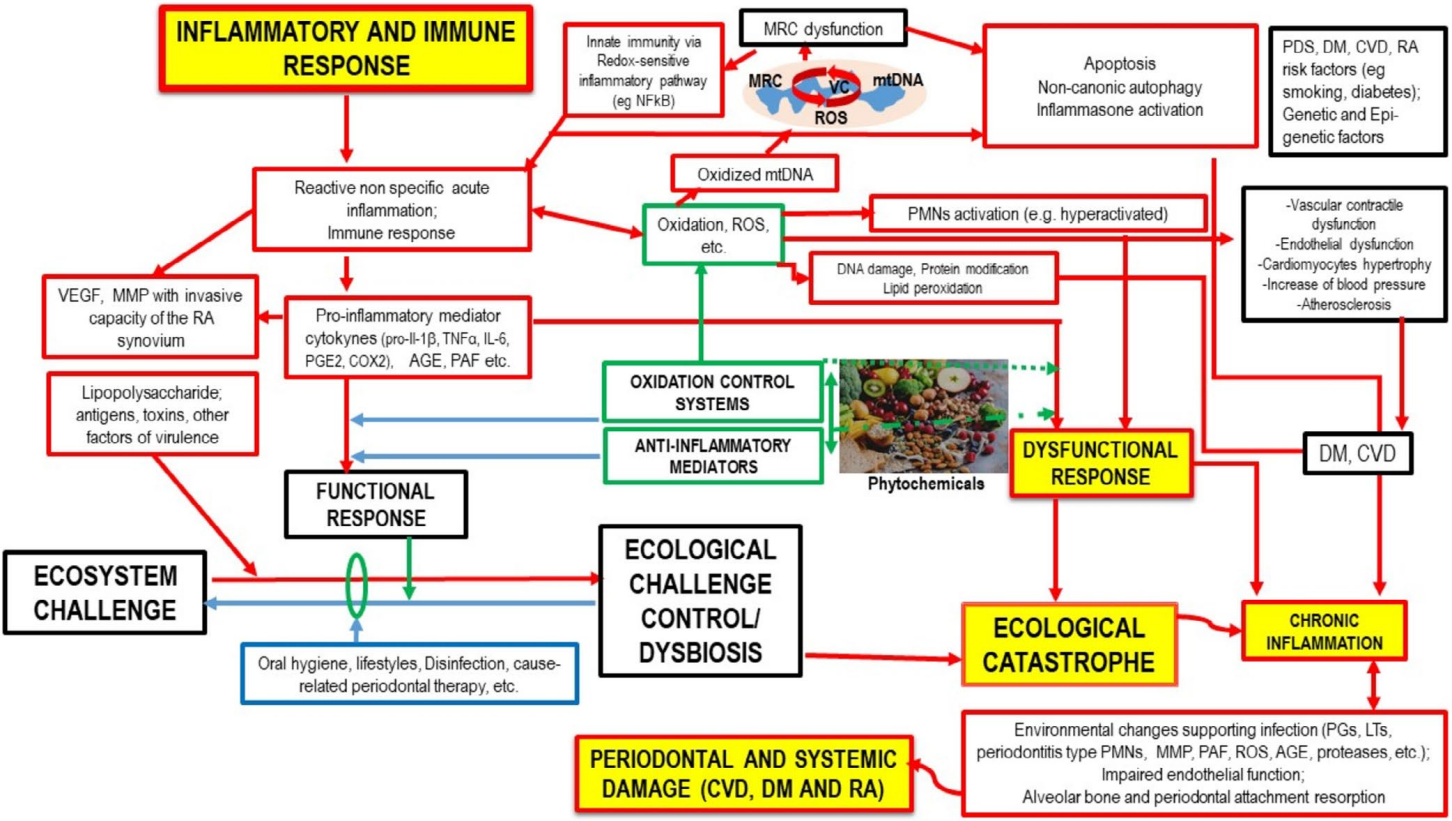
Inflammation and oxidation: pathogenesis of PDS and systemic damages. Legend: Red arrows indicate a prevailing proinflammatory activity, whereas blue arrows show a prevailing proresolving activity and green arrows indicate modulation effects. Red borders describe inflammatory phases, blue borders indicate proresolving phases and black borders symbolize the pathophysiological environment in which the different phases of inflammation occur, green borders show modulation effects. AGE, advanced glycation end‐product; COX, cyclooxygenase; CVD, cardiovascular diseases; DM, diabetes mellitus; LTs, leukotrienes; MMP, matrix metalloproteinase; MRC, mitochondrial respiratory chain; NF‐kB, nuclear factor kB; PAF, platelet‐activating factor; PDS, periodontitis; PG, prostaglandins; PMNs, polymorphonuclear neutrophils; RA, rheumatoid arthritis; ROS, reactive oxygen species; TNFα, tumor necrosis factor α; VC, vitious cycle; and VEGF, vascular endothelial growth factor.

Many chronic diseases have inflammation as a common pathogenesis, including periodontitis (PDS), cardiovascular diseases (CVD), type II diabetes mellitus (DM), and rheumatoid arthritis (RA), which are physiopathologically associated with each other (Bertoldi et al. 2024; Sanz et al. 2020b; Rak et al. 2024; Păunică et al. 2023) (Figures 1 and 2). The temporary upregulation of inflammation, which occurs when a threat is present and ceases once the threat is resolved, characterizes a normal inflammatory response (Netea et al. 2017; Slavich 2015; Borella et al. 2022).

The presence of determined biological, psychosocial, and environmental factors is linked to the lack of resolution of acute inflammation and, in turn, to the progression of a state of variable‐grade systemic chronic inflammation (Figure 1) (Bertoldi et al. 2013; Leuti et al. 2019).

The oxidative stress results in close relation with inflammation, and is among the major suspects of the shift toward chronic inflammation (de Gaetano et al. 2021). Oral tissues are constantly subjected to reactive oxygen species (ROS) (de Gaetano et al. 2021) (Ambati et al. 2017). The principal source of free radicals in the oral cavity is physiological metabolism, as mitochondrial respiration inevitably leads to the production of ROS (de Gaetano et al. 2021). However, ROS can also be generated by exposure to external factors, including oral microbiota, air pollution, cigarette smoke, alcohol, ionizing and ultraviolet radiation, drugs, medicines, dental materials, and dental treatments (Figure 2) (Ambati et al. 2017).

All the inflammatory processes share a common mechanism, which triggers when a threat is present (Figures 1 and 2) (Monsarrat et al. 2016; de Biasi et al. 2022). In this context, describing the pathways of onset and regulation of inflammation can highlight some promising avenues for research and intervention in diseases with inflammatory pathogenesis.

The oxidation role in the pathogenesis of PDS and several diseases linked to chronic inflammation, such as CVD, DM, and RA, has been one of the most neglected pathogenic aspects, only recently being considered. To address this knowledge gap, a narrative review approach was chosen to gather evidence evaluating the active role of periodontal therapy and dietary lifestyle in switching off the chronic inflammation, oxidation restraining, and pathologies connected to them, such as CVD, type II DM, and RA.

## 2 Methods

This narrative review considers preclinical and clinical studies in English, with no restrictions on the year of publication. The study focuses on the following questions:

- What is the burden of PDS?
- What is the relationship between inflammation and oxidation?
- What could be the joint role of inflammation in PDS, CVD, DM, and RA pathogenesis?
- What is the therapeutic effect of periodontal therapy (NSPT) in inflammation resolving?
- What is the therapeutic effect of polyunsaturated fatty acids (PUFAs) and plant‐derived polyphenols in oxidation and inflammation resolving?

**FIGURE 3:**
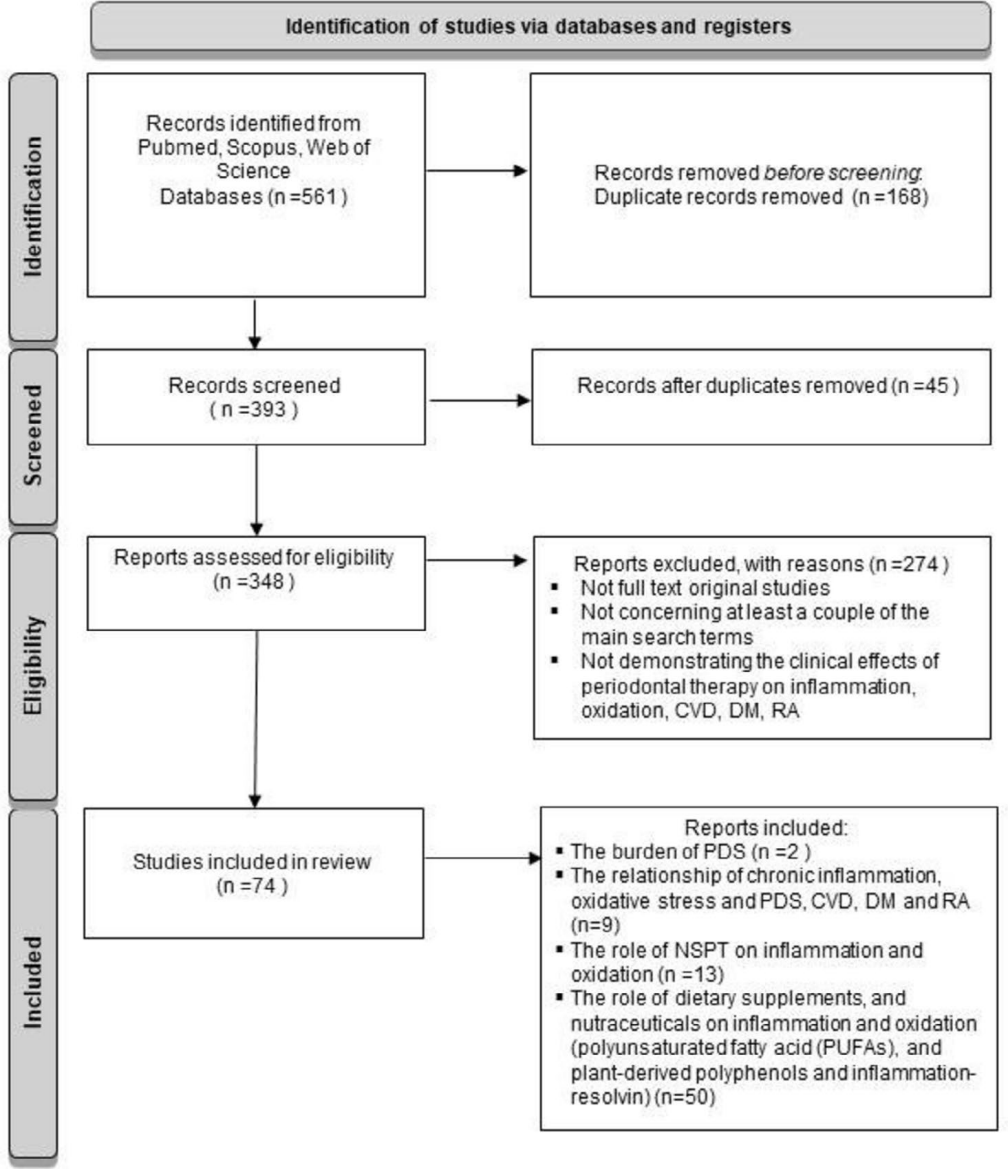
Narrative review flow diagram illustrating the systematic process of identifying studies from various databases, screening them, assessing their eligibility, and including those that are suitable for the research purpose.

The electronic databases Medline (via PubMed), Scopus, and Web of Science were analyzed for eligible studies in periodontology, cardiology, and immunology. The main search terms were “inflammation.”, “oxidative stress,” “periodontitis,” “cardiovascular diseases,” “diabetes mellitus,” “rheumatoid arthritis,” “non‐surgical periodontal therapy/treatment,” “nourishment,” and “nutrient.” Afterward, the title and abstract of acquired articles were manually examined for relevance to the research criteria. The inclusion criteria were the following: (i) full‐text original studies; (ii) full‐text original studies concerning all at once or, at least, a couple of the main search terms research; (iii) articles demonstrating the clinical effect(s) of periodontal therapy on inflammation and oxidation; (iv) articles indicating the clinical effect(s) of nourishment on PDS; and (v) articles indicating the clinical effect(s) of periodontal therapy or/and nourishment on CVD, DM, and RA. Figure 3 shows the literature selection. The references contained in original studies and review articles were also investigated for potential inclusion. No further inclusion criteria were considered.

## 3 Results and Discussion

### 3.1 The Burden of PDS

**FIGURE 4:**
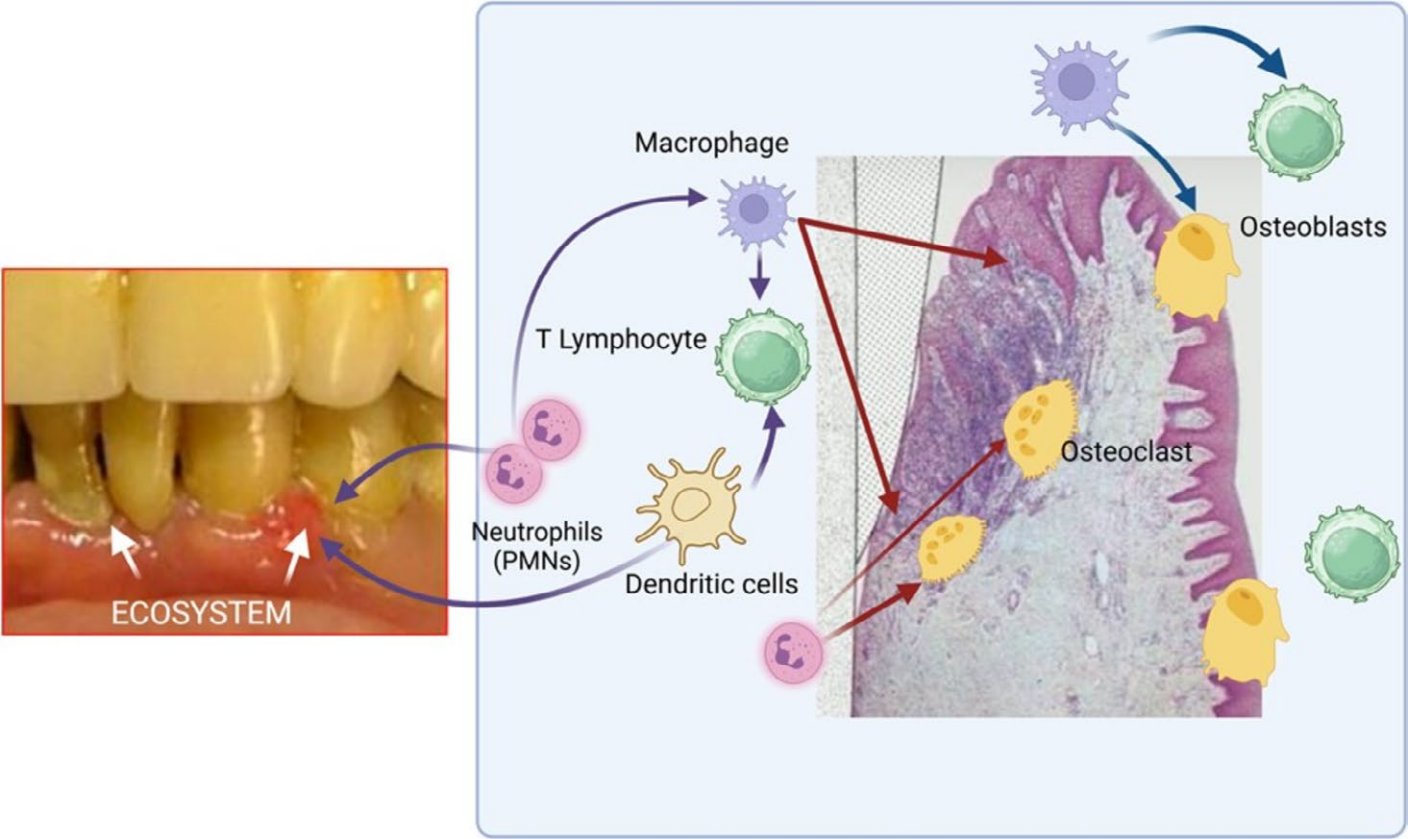
Gingivitis and periodontal damage formation. Host immune microenvironmental cell regulation in PDS. The periodontal soft and hard tissues are remodeled by the involvement of immune cells, including a large number of neutrophils, macrophages, dendritic cells, T cells, and host stem cells.

**FIGURE 5:**
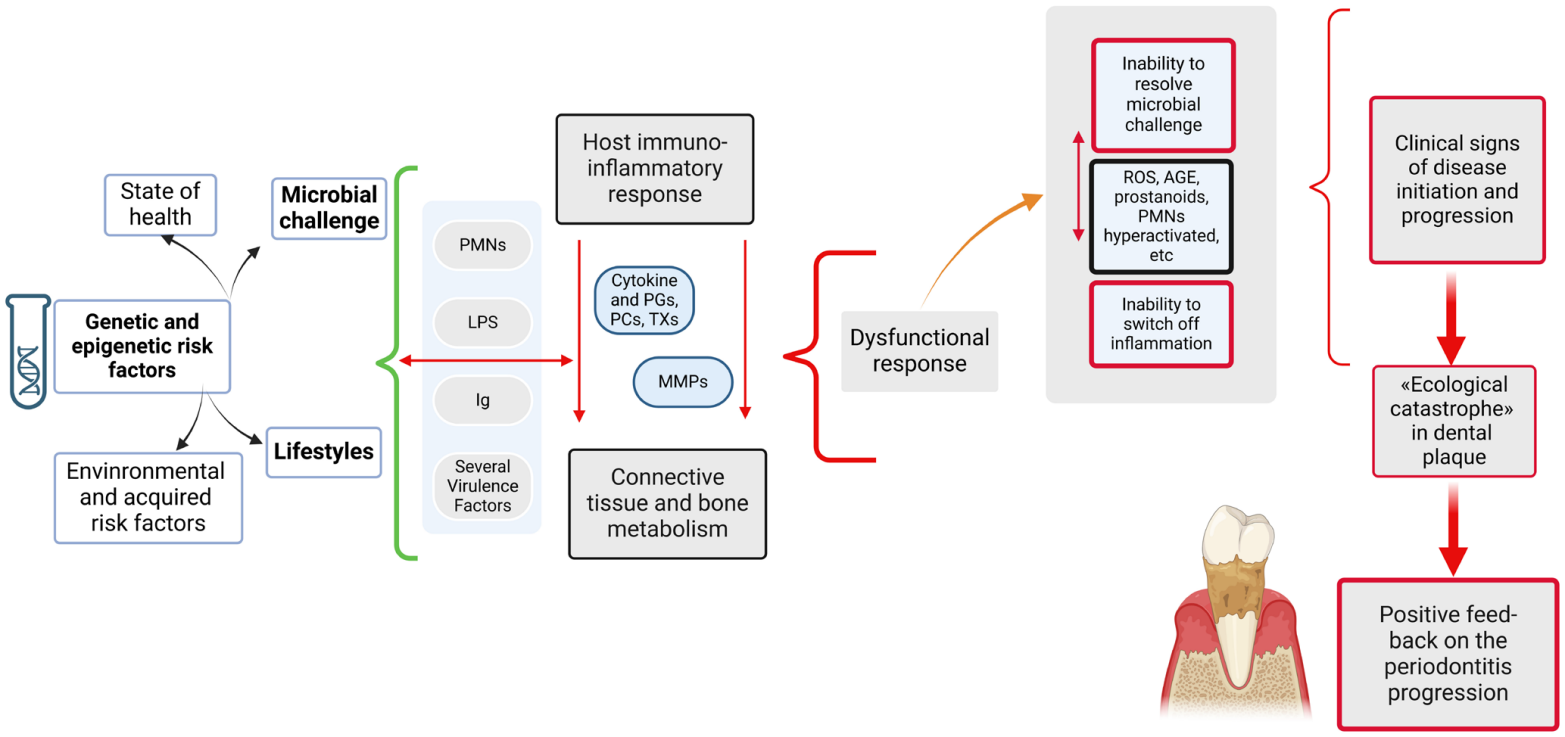
Pathogenesis of periodontitis; microbiota challenge plays a pathogenic key role in PDS progress. Legend: Red arrows and red braces indicate a prevailing proinflammatory activity and green brace indicates modulation effects. Red borders describe inflammatory phases, and black borders symbolize pathophysiological environments and activities. AGE, advanced glycation end‐product; Ig, immunoglobulins; LPS, lipopolysaccharides; MMPs, matrix metalloproteinases; PCs, prostacyclins; PGs, prostaglandins; PMNs, polymorphonuclear neutrophils; ROS, reactive oxygen species; and TXs, thromboxanes.

If untreated, PDS produces periodontal defects that are not spontaneously reversible, leading to tooth loss (Figures 4 and 5) (Bertoldi et al. 2024; Sanz et al. 2020a).

Kassebaum et al. reported that severe periodontal disease (PDS) ranks as the sixth most common pathological condition globally, affecting 10.8% of the population, which translates to approximately 743 million individuals worldwide (Kassebaum et al. 2014). According to the National Health and Nutrition Examination Survey (NHANES), the prevalence of PDS among adults aged 30 and older was 47.2% from 2009 to 2010, equating to around 64.7 million adults in the United States. This figure rose to 64% among those aged 64 and older. Additionally, a follow‐up study from 2009 to 2012 revealed that 8.9% of US adults aged 30 and over experienced severe PDS. The economic impact of dental diseases was estimated at $442 billion, with $298 billion attributed to direct treatment costs and $144 billion to indirect costs due to productivity losses. Of the indirect costs, about 37.5% ($53.99 billion) were linked to PDS (Bertoldi et al. 2024). Data from the Global Burden of Disease Study (1990–2010) indicated that oral conditions were highly prevalent in 2010, affecting 3.9 billion people globally, with a significant increase in the prevalence and burden associated with severe PDS, as measured by disability‐adjusted life years (DALYs) (Bertoldi et al. 2024). Given the rising average age and ongoing growth of the global population, these figures are likely to continue increasing. However, aging alone does not necessarily lead to pathological loss of periodontal attachment in otherwise healthy elderly individuals (Bertoldi et al. 2024).

### 3.2 Relationship of Chronic Inflammation, Oxidative Stress, and PDS, CVD, DM, and RA

In a recent systematic review of registered trials, Monsarrat et al. (de Gaetano et al. 2021) identified 56 systemic diseases that are hypothesized to be associated with periodontal diseases, accounting for nearly 2% of diseases indexed in the MeSH vocabulary.

**FIGURE 6:**
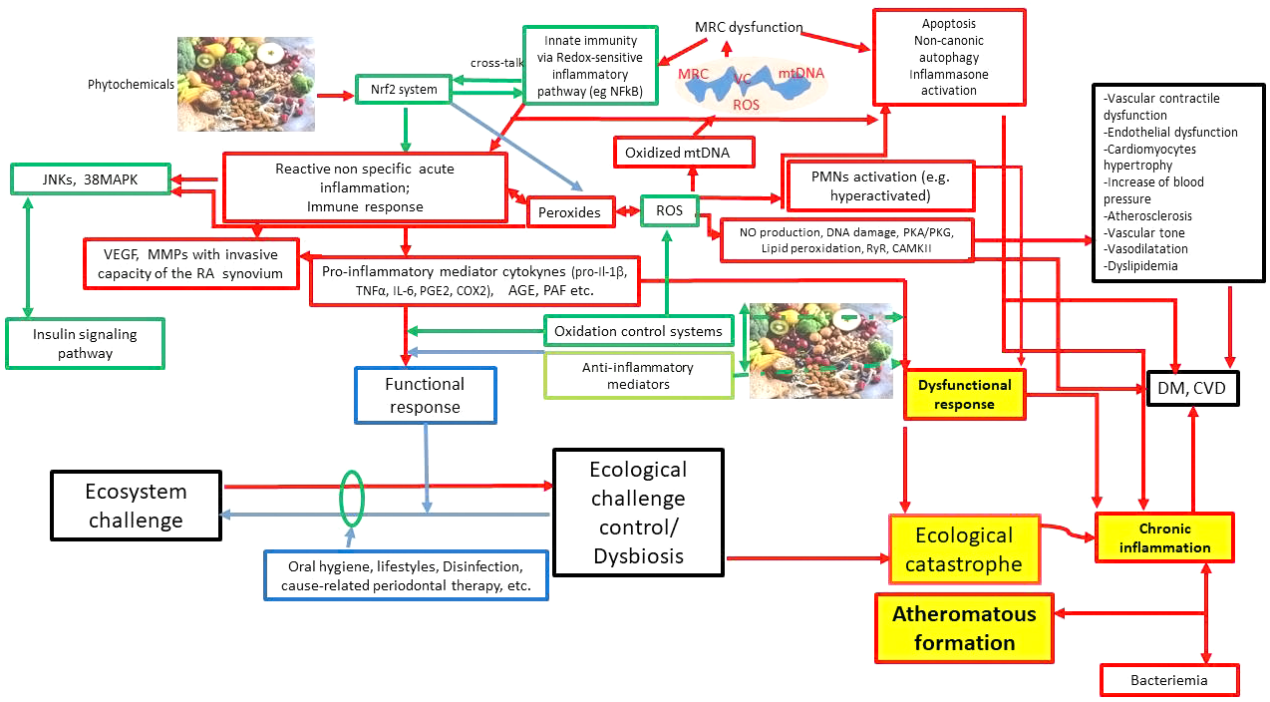
Inflammation and oxidation: pathogenesis of CVD. Red arrows indicate a prevailing proinflammatory activity, whereas blue arrows show a prevailing proresolving activity and green arrows indicate modulation effects. Red borders describe inflammatory phases, blue borders indicate proresolving phases, black borders symbolize the pathophysiological environment in which the different phases of inflammation occur, and green borders show modulation effects. Red braces indicate a prevailing proinflammatory activity and green brace indicates modulation effects. AGE, advanced glycation end‐product; CAMKII, a/calmodulin‐dependent kinase II; COX, cyclooxygenase; CVD, cardiovascular diseases; DM, diabetes mellitus; IL, interleukine; JNK, c‐Jun N‐terminal kinase; LTs, leukotrienes; MMP, matrix metalloproteinase; MRC, mitochondrial respiratory chain; NFERF2, nuclear factor erythroid 2–related factor 2; NF‐kB, nuclear factor kB; NO, nitric oxide; p38 MAPK, p38 mitogen‐activated protein kinase; PAF, platelet‐activating factor; PDS, periodontitis; PG, prostaglandins; PKA/PKG, protein kinase A/G; PMNs, polymorphonuclear neutrophils; RA, rheumatoid arthritis; ROS, reactive oxygen species; RyR, ryanodine receptor; TNFα, tumor necrosis factor α; VC, vitious cycle; and VEGF, vascular endothelial growth factor.

**FIGURE 7:**
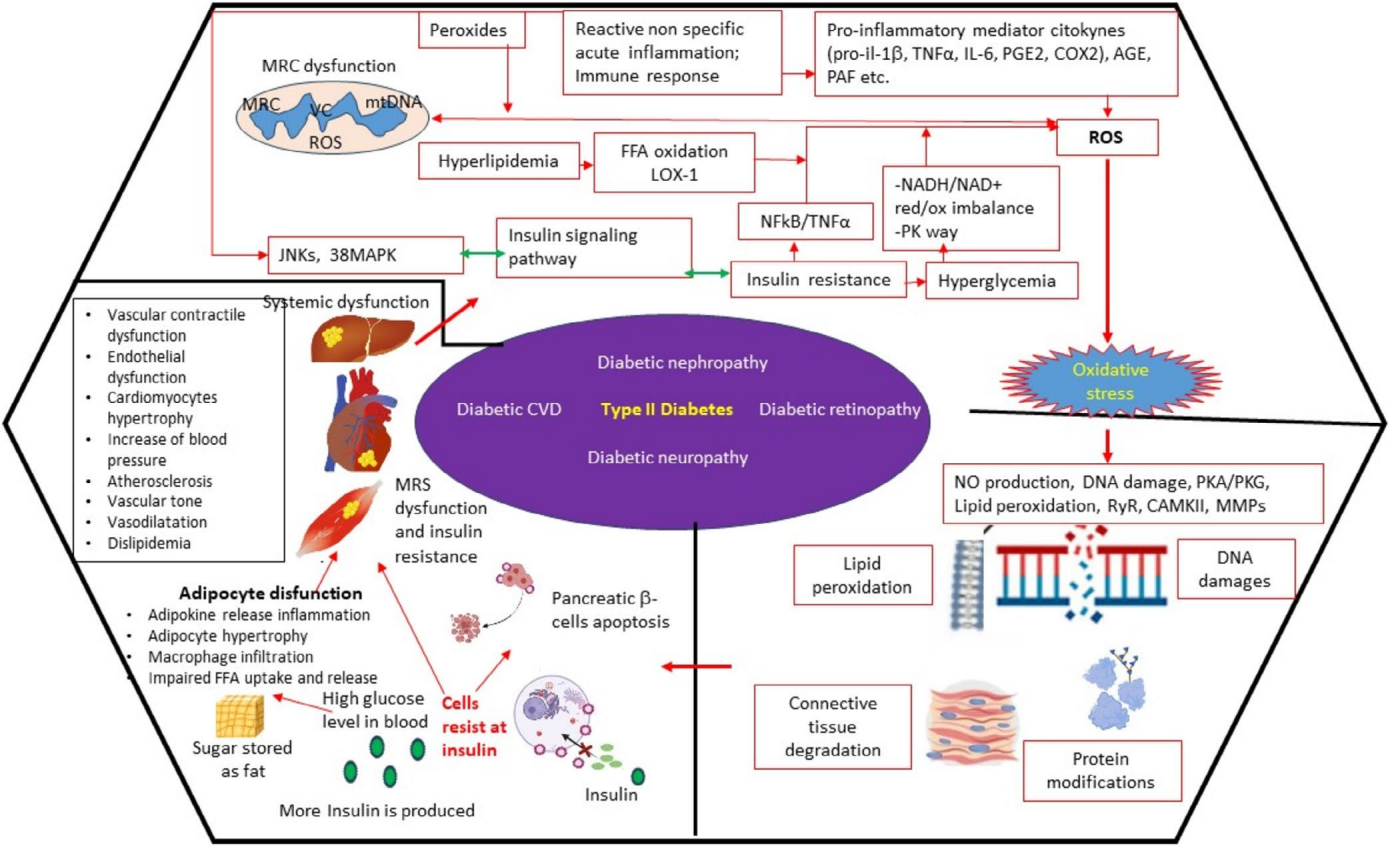
Inflammation and oxidation: pathogenesis of DM. Legend: Red arrow represents activation and green arrows represent modulation. Red borders describe inflammatory phases and black borders symbolize the pathophysiological environment in which the different phases of inflammation occur. AGE, advanced glycation end‐product; CAMKII, a/calmodulin‐dependent kinase II; COX, cyclooxygenase; CVD, cardiovascular diseases; DM, diabetes mellitus; FFA, free fatty acids; IL, Interleukine; JNK, c‐Jun N‐terminal kinase; LOX‐1, lectin‐like ox‐LDL receptor‐1; LTs, leukotrienes; MMP, matrix metalloproteinase; MRC, mitochondrial respiratory chain; NADH/NAD+, nicotinamide adenin dinucleotide reduce/oxidated from; NFERF2, nuclear factor erythroid 2–related factor 2; NF‐kB, nuclear factor kB; NO, nitric oxide; p38 MAPK, p38 mitogen‐activated protein kinase; PAF, platelet‐activating factor; PDS, periodontitis; PG, prostaglandins; PKA/PKG, protein kinase A/G; PMNs, polymorphonuclear neutrophils; RA, rheumatoid arthritis; ROS, reactive oxygen species; RyR, ryanodine receptor; TNFα, tumor necrosis factor α; VC, vitious cycle; and VEGF, vascular endothelial growth factor.

**FIGURE 8:**
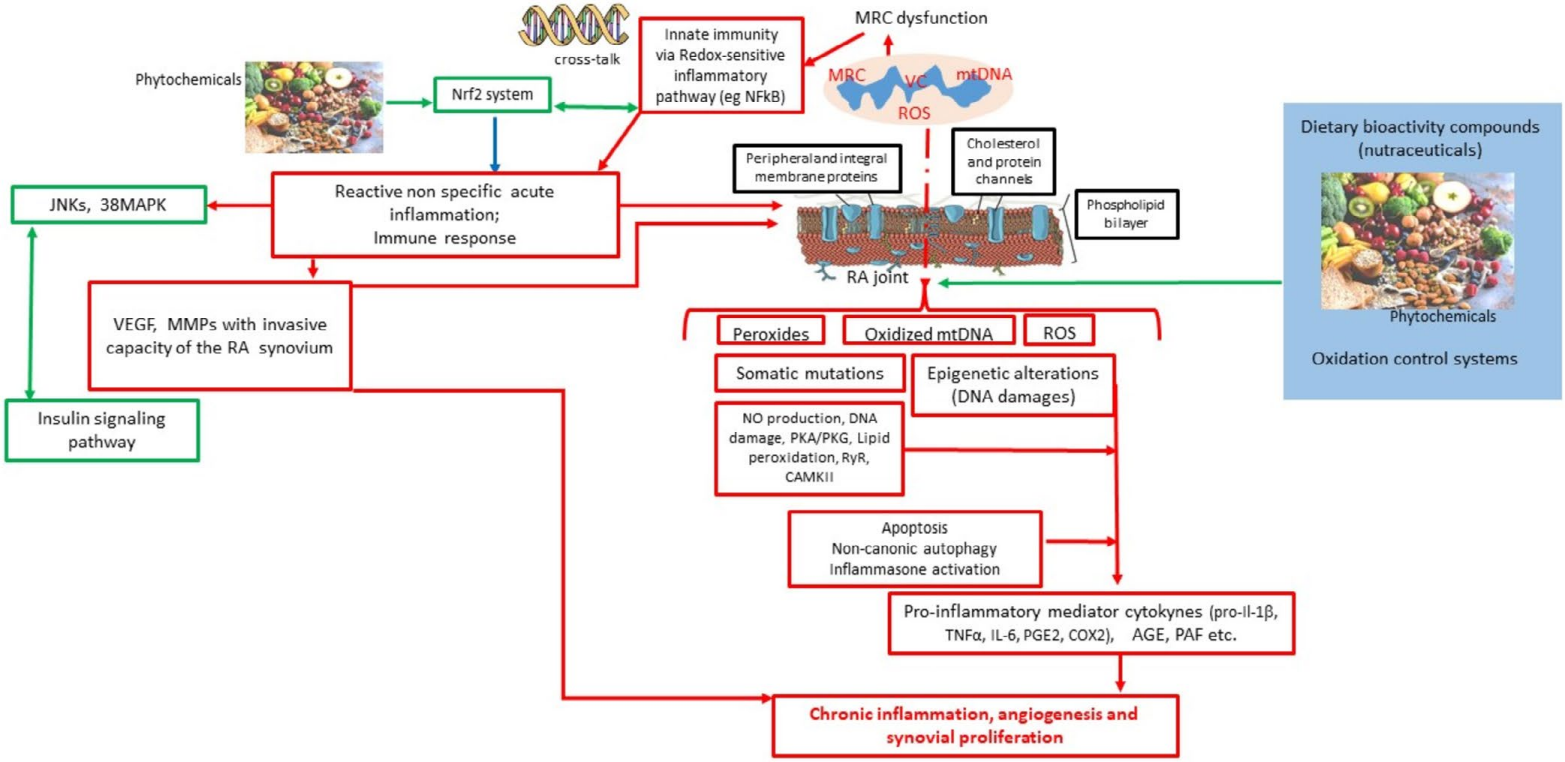
Inflammation and oxidation: pathogenesis of RA. Legend: Red arrows indicate a prevailing proinflammatory activity, whereas blue arrows show a prevailing proresolving activity. Red borders describe inflammatory phases, blue borders indicate proresolving phases, and black borders symbolize the pathophysiological environment in which the different phases of inflammation occur. AGE, advanced glycation end‐product; CAMKII, Ca/calmodulin‐dependent kinase II; COX, cyclooxygenase; CVD, cardiovascular diseases; DM, diabetes mellitus; IL, interleukine; JNK, c‐Jun N‐terminal kinase; LTs, leukotrienes; MMP, matrix metalloproteinase; MRC, mitochondrial respiratory chain; NFERF2, nuclear factor erythroid 2–related factor 2; NF‐kB, nuclear factor kB; NO, nitric oxide; p38 MAPK, p38 mitogen‐activated protein kinase; PAF, platelet‐activating factor; PDS, periodontitis; PG, prostaglandins; PKA/PKG = protein kinase A/G; PMNs = polymorphonuclear neutrophils; RA = rheumatoid arthritis; ROS = reactive oxygen species; RyR = ryanodine receptor; TNFα = tumor necrosis factor α; VC = vitious cycle; and VEGF = vascular endothelial growth factor.

ROS could be conjectured as a “double ax” because it has a key role in removing infecting pathogens, but beyond a certain concentration, ROS results in being toxic to host cells and tissues. (Bertoldi et al. 2013) (Johnstone et al. 2007). Elevated levels of oxidative stress (i.e., elevated levels of ROS) and the severity of PDS were proved to be in direct correlation (Novakovic et al. 2014; Chapple et al. 2007) (Figures 6, 7, 8).

PDS is correlated with a “hyperactivated” PMN phenotype, which is characterized by the overproduction of proteases and ROS (Figures 2, 5 and 7) (Fine et al. 2016).

PMNs are considered the primary sources of ROS in PDS, followed by monocytes/macrophages (Leuti et al. 2019).

**FIGURE 9:**
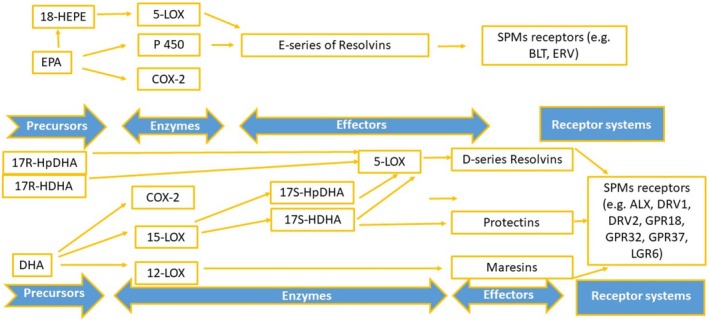
Specialized proresolving mediators (SPMs). Legend: 17R‐HDHA, 17R‐Hydroxy‐docosahexaenoic acid; 17R‐HpDHA, 17R‐hydroperoxy‐docosahexaenoic acid; 17S‐HDHA, 17S‐hydroxy‐docosahexaenoic acid; 17S‐HpDHA, 17S‐hydroperoxy‐docosahexaenoic acid; 18‐HEPE, 18‐hydroxyeicosapentaenoic acid; 5‐LOX, 5‐ lipoxygenase; COX, cyclooxygenase; DHA, docosahexaenoic acid; EPA, eicosapentaenoic acid; and P450, cytochrome P 450.

We already know that PMNs play a relevant role in the induction, control, and even resolution of the acute phase of inflammation through their immune function, interaction with phagocytosis, their lysis, and also the release of inflammatory specialized proresolving molecules (SPMs), such as lipoxin, resolvins (Rv), protectins, and maresins (Figures 1 and 9) (Leuti et al. 2019).

The oxidative imbalance triggers proinflammatory mechanisms and osteoclastogenesis, leading to bone ridge loss, which is a key diagnostic feature in the staging not only of PDS but also of arthritis (Figures 4, 5 and 8). The loss of destruction of mineralized and nonmineralized tissues happens concomitantly; specific proinflammatory networks are correlated with damage either to soft tissue or hard and soft tissues. Besides, the large amount of different matrix metalloproteinases (MMPs) released at the site of the infective challenge during the immune response, along with the effect of ROS on fibroblasts, favors the disruption of bone and connective tissue matrices (Figures 6, 7 and 8) (Hienz et al. 2015).

**FIGURE 10:**
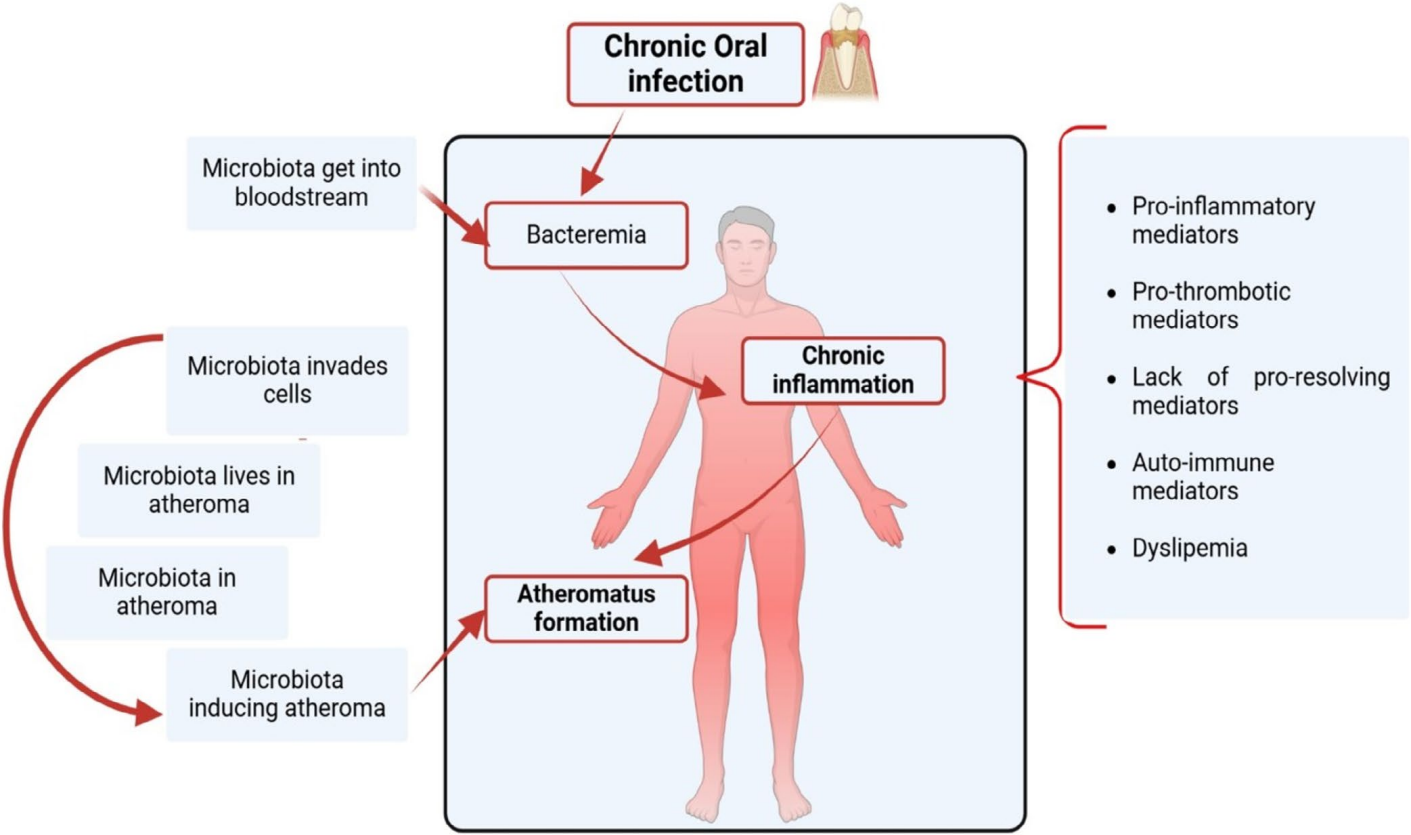
Genesis of atherotrombotic lesions.

However, the biological pathways that we have described are not exclusively relevant to PDS (Figures 2, 6, 7 and 8). The oxidative imbalance also affects a wider variety of inflammatory conditions and behaviors that trigger oxidative stress. Some systemic diseases, such as CVD (Figures 6 and 10), type 2 DM (Figure 7), and RA (Figure 8), are included in this range and show correlations with oral conditions (Fine et al. 2016)–(Sczepanik et al. 2020).

### 3.3 The Role of NSPT on Inflammation and Oxidation

PDS patients show a higher tendency for CVD, DM, and RA (Sanz et al. 2020b)–(Păunică et al. 2023).

In particular, PDS, CVD, type II DM, and RA share similar inflammatory patterns, and a bidirectional relationship has been found between PDS and these same diseases (Bertoldi et al. 2024; Păunică et al. 2023; Engebretson and Kocher 2013). Analysis of major intervention studies with long‐term follow‐up and large sample sizes shows that NSPT has clinically beneficial effects on both subclinical and clinical CVD (Bertoldi et al. 2024).

Mainly, NSPT can significantly improve glycemic control in periodontal patients. After periodontal treatment, Sun et al. (2011) a significant mean reduction of hemoglobin A1c (HbA1c) levels in type 2 diabetes patients (Sun et al. 2024). Sun et al. highlighted a significant mean reduction of hemoglobin A1c (HbA1c) levels in type 2 diabetes patients (Sun et al. 2024). Specifically, the positive impact of periodontal therapy on glycemic control was significantly more pronounced in studies with a higher baseline HbA1c level compared to those with a lower baseline HbA1c level. In a recent prospective cohort study, Mizutani et al. (Mizutani et al. 2024) provided compelling evidence of a significant improvement in periodontal indices following intensive diabetes care.

NSPT can significantly reduce the severity of RA in patients with Ra and PDS (Nguyen et al. 2021; Popoca‐Hernández et al. 2024). Additionally, Zhang et al. (Zhang et al. 2022) reported that antirheumatic agents have a beneficial impact on the periodontal health of patients with RA and PDS.

The association between oxidative stress and PDS was also confirmed by Martínez‐Herrera et al. (Martínez‐Herrera et al. 2020) and Nisha et al. (Nisha et al. 2023), highlighting the positive therapeutic effects of NSPT on antioxidant activity.

NSPT can reduce chronic gingival and systemic inflammation, after a short initial phase of increase. Oxidative stress related to the microbiota and oral inflammation is also effectively reduced, but periodontal therapy has the limit of being performed only locally. Therefore, a further antioxidant systemic therapy may provide helpful additional systemic therapeutic benefits that periodontal therapy alone could not achieve (Bertoldi et al. 2024; Martínez‐Herrera et al. 2020; Nisha et al. 2023).

### 3.4 The Role of Dietary Supplements and Nutraceuticals on Inflammation and Oxidation

**FIGURE 11:**
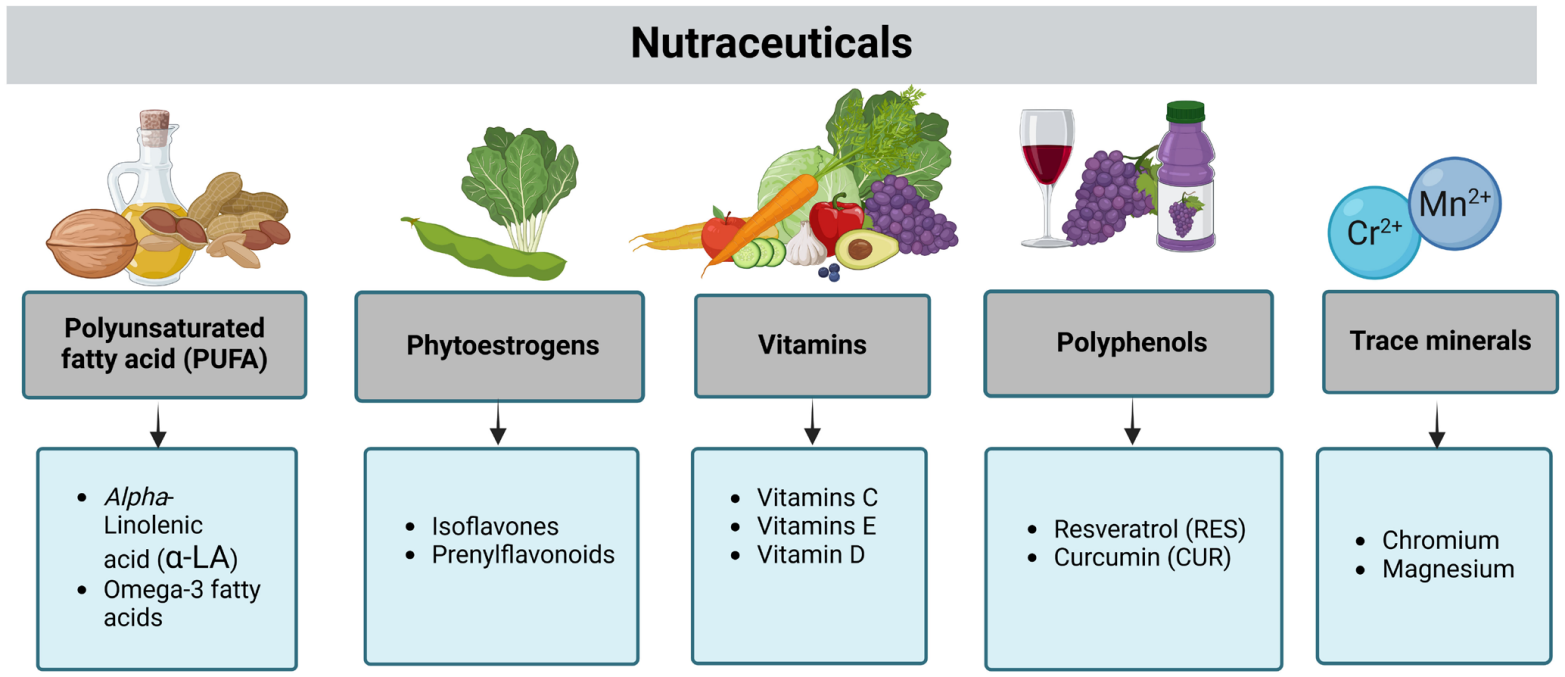
Nutraceuticals. Classification of nutraceuticals based on key bioactive compounds and dietary sources: polyunsaturated fatty acids (PUFA), phytoestrogens, vitamins, polyphenols, and trace minerals.

The use of dietary supplements and nutraceuticals represents a very promising branch of therapeutic development. These compounds include PUFAs, plant‐derived polyphenols, plant flavonoids, phytoestrogens, antioxidant vitamins such as vitamins C, D, and E, flavonoids, dietary fibers, and more (Figure 11).

#### 3.4.1 PUFAs

**FIGURE 12:**
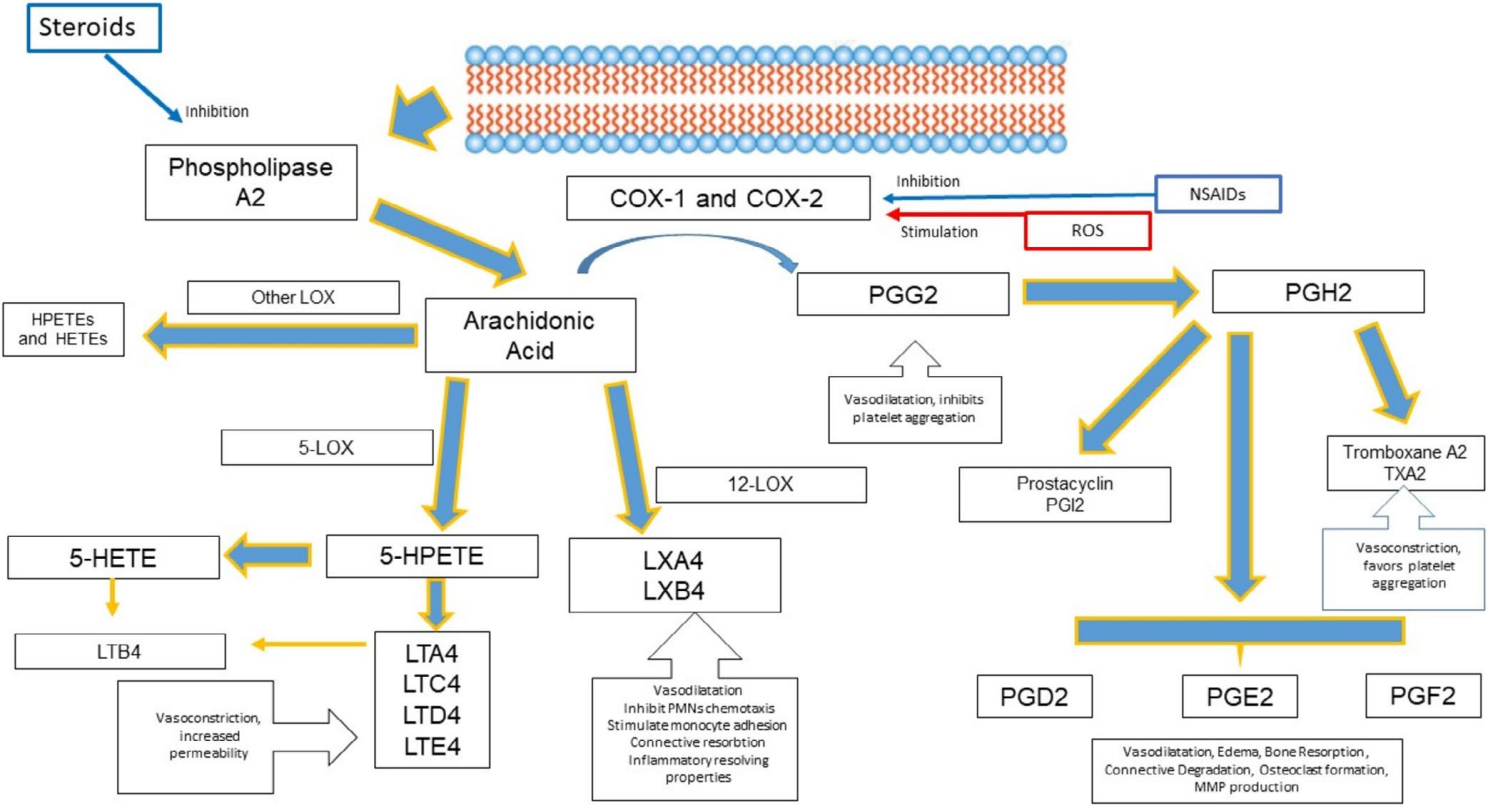
Arachidonic acid cascade. Legend: 5‐HETE, 5‐Hydroxyeicosatetraenoic acid; 5‐HPETE, 5‐hydroperoxyeicosatetraenoic acid; 5‐LOX, 5‐lipoxygenase; COX, cyclooxygenase; LT, leukotriene; LX, lipoxin; MMP, matrix metalloproteinase; NSAID, nonsteroidal anti‐inflammatory drugs; PG, prostaglandin; PMNs, polymorphonuclear neutrophils; and ROS, reactive oxygen species.

**FIGURE 13:**
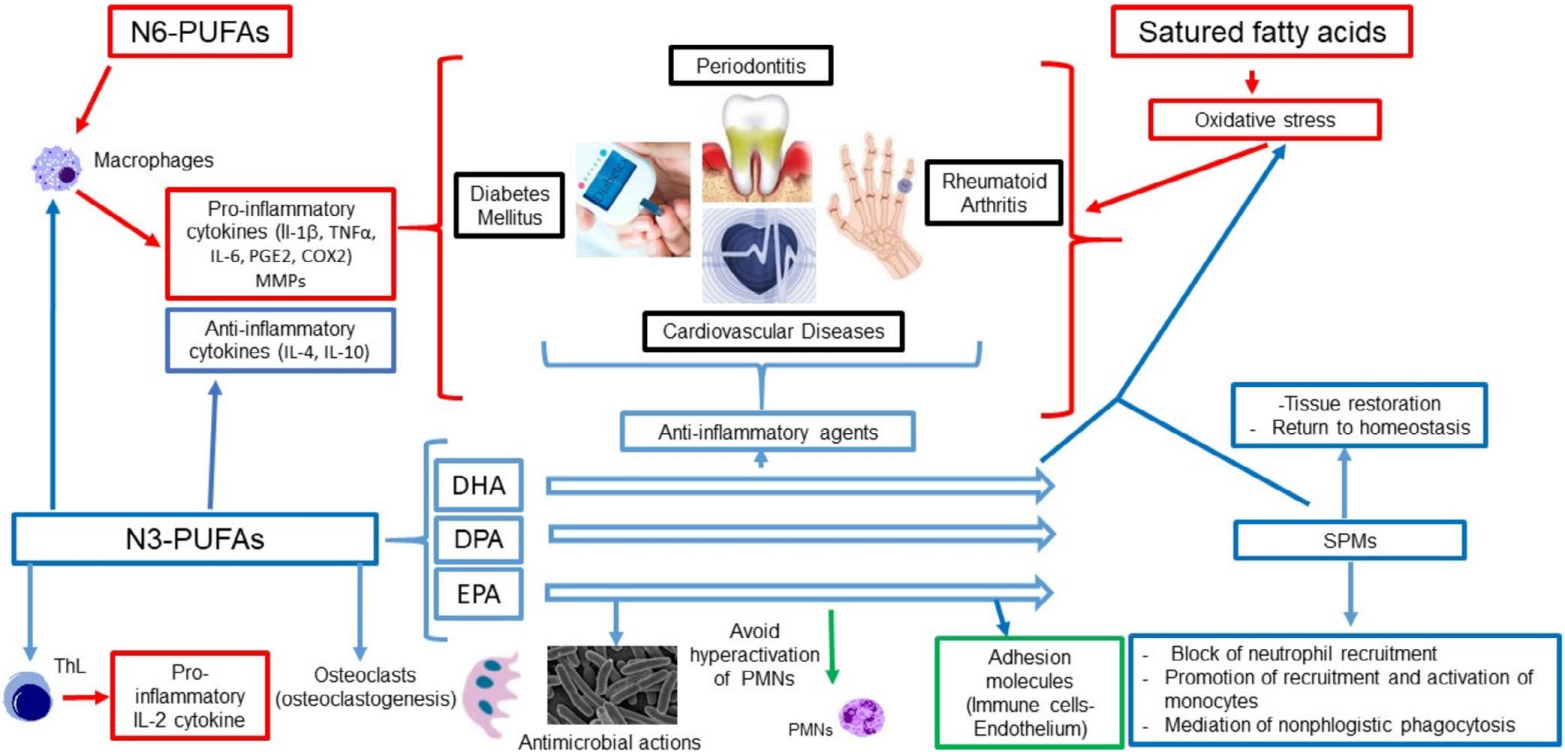
Polyunsaturated fatty acids (PUFAs) effects. Legend: Red arrow represents activation, blue arrow represents inhibition, and green arrow represents modulation. Red borders describe inflammatory phases, black borders symbolize the pathophysiological environment in which the different phases of inflammation occur, green borders show modulation effects, and blue borders indicate proresolving phases. Red braces indicate a prevailing proinflammatory activity and blue braces indicate anti‐inflammatory activity. DHA, docosahexaenoic acid; DPA, docosapentaenoic acid; EPA, eicosapentaenoic acid; IL, interleukine; LT, leukotriene; LX, lipoxin; MMP, matrix metalloproteinase; PG, prostaglandin; PMNs, polymorphonuclear neutrophils; PMNs, polymorphonuclear neutrophils; and SPMs, specialized proresolving mediators.

PUFAs have been widely studied for their inflammation‐modulating properties. Two main groups of PUFAs exist: the n‐6 (or ω‐6) and the n‐3 (or ω‐3). The key long‐chain PUFAs include the n‐3 derivatives docosahexaenoic acid (DHA), eicosapentaenoic acid (EPA), and docosapentaenoic acid (DPA), as well as the n‐6 derivative arachidonic acid (AA) (Figure 12). Omega‐3 PUFAs mitigate the effect of AA‐derived eicosanoids (n‐6 PUFAs) by exhibiting anti‐inflammatory activity. Omega‐3 fatty acids, specifically EPA and DHA, help lower the production of classic inflammatory cytokines. Additionally, a significantly high n‐6/n‐3 ratio could be a key factor in the development of various diseases (Figures 9, 12, and 13).

EPA and DHA compete with AA for the cyclooxygenase (COX) and lipoxygenase (LOX) pathways, leading to a decreased production of highly active AA metabolites. This competition results in the generation of series‐3 prostaglandins (PG3) and series‐5 leukotrienes (LT5), both of which have anti‐inflammatory properties. Additionally, EPA and DHA have been shown to modify the cellular functions of PMNs, regulate lymphocyte proliferation, and boost the body's natural antioxidant defenses (Figure 13) (Sczepanik et al. 2020; Kesavalu et al. 2007; Heo et al. 2022).

Growing evidence suggests that polyunsaturated fatty acids (PUFAs) play a beneficial role in managing type II diabetes mellitus (DM) (Wang et al. 2024), CVD, chronic inflammation associated with rheumatoid arthritis (RA) (Parolini 2023), and PDS (Panezai and van Dyke 2023). A recent meta‐analysis of 14 randomized controlled trials found that omega‐3 PUFAs significantly reduced the risk of major adverse cardiovascular events, cardiovascular death, and myocardial infarction, although they did not have a notable impact on all‐cause mortality (Shen et al. 2022). Additionally, systematic reviews and meta‐analyses indicate that omega‐3 PUFAs, when used alongside nonsurgical periodontal therapy (NSPT), lead to significantly better outcomes for PDS patients (Savran and Sağlam 2024). However, variations in endpoints, observation periods, sample sizes, inclusion criteria, and dosages of EPA and DHA limit the comparability of different studies (Sanz et al. 2020a).

#### 3.4.2 Plant‐Derived Polyphenols

Plant‐derived polyphenols have been considered antioxidants, acting directly against ROS overproduction and in reestablishing the redox balance. The most investigated polyphenols are curcumins (CURs) followed by resveratrol (RES), quercetin (QUE), epigallocatechin gallate (EGCG), and apigenin (AP) because of their potential anticancer effects, alone or in combination with chemotherapeutic drugs (Jakobušić Brala et al. 2023).

#### 3.4.3 CURs

CURs are a blend of three curcuminoids derived from polyphenols and are regarded as effective anti‐inflammatories (Jakobušić Brala et al. 2023). However, CURs and CURs derivatives and analogs have also been studied for their antimicrobial and antioxidant properties, as they have several polyphenols. Besides, they are also deemed very promising antitumor agents, modulating the tumor microenvironment, including factors such as acidic pH, elevated ROS levels, and hypoxic conditions.

CURs seem to exert protective effects in age‐related cellular senescence and may help prevent the decline of vascular functions associated with aging. Numerous studies suggest that CUR could be a promising nutraceutical treatment for age‐related CVD. In terms of secondary prevention, CURs could play a significant role in safeguarding cardiomyocytes from injury caused by ischemia and hypoxia, inhibiting myocardial hypertrophy and fibrosis, enhancing ventricular remodeling, mitigating drug‐induced myocardial damage, improving diabetic cardiomyopathy, alleviating vascular endothelial dysfunction, preventing foam cell formation, and reducing the proliferation of vascular smooth muscle cells (Cox et al. 2022; Yang et al. 2024).

CURs show antidiabetic effects through complex pharmacological mechanisms that help lower the hyperglycemia associated with diabetes mellitus. Given these findings, CURs could be considered a potential option for the pharmacotherapeutic management of diabetic patients (Quispe et al. 2022).

CURs are beneficial for treating RA, as they can help reduce inflammation levels and alleviate clinical symptoms in patients (Kou et al. 2023). Nevertheless, CURs appear to have efficient antibacterial activity and inhibit infection and biofilm formation. They exert unique immunosuppressive properties, reduce proinflammatory responses, and encourage the development of regulatory T cells, thereby helping to suppress autoimmunity (Figures 6 and 8) (Asteriou et al. 2018). Consequently, CURs may help mitigate alveolar bone loss and prevent matrix degradation, bone resorption, and the release of free radicals, all of which are significant factors in the progression of PDS (Guru et al. 2017). This pathway is a key mechanism through which CURs promote favorable clinical outcomes. Current evidence suggests that CUR is effective in periodontal therapy compared to NSPT alone. Additionally, CUR is a natural herbal remedy with minimal side effects, making it a strong candidate for adjunctive treatment in periodontal disease (Zhang et al. 2022). Joint diseases can develop in previously healthy individuals, often characterized by low levels of circulating antioxidants, which indicate a pathogenic role of heightened oxidative stress in the onset of joint diseases, including RA and PDS (Lee et al. 2006).

#### 3.4.4 RES

RES could have antitumor, antimicrobial, cardioprotective, and vasoprotective properties and could improve control of type 2 diabetes and RA (Valookaran et al. 2022; Zare Javid et al. 2017). RES alleviates the inflammatory effects and inhibits the progression of RA by suppressing excessive ROS during RA development. Besides, RES could provide an innovative approach to treating RA and PDS. RES decreases inflammation in human gingival tissue and enhances the stemness of gingival mesenchymal stem cells. Cell experiments revealed that RES reduces the expression of proinflammatory mediators, alleviates inflammation, promotes the proliferation and osteogenic differentiation of gingival mesenchymal stem cells, and enhances their immunomodulatory capabilities (Jiang et al. 2023).

Overall, combining RES with NSPT may be advantageous in improving clinical parameters and reducing inflammation in patients with PDS (Nikniaz et al. 2023).

Although relying solely on a diet rich in RES to stay healthy or prolong life may not be the right choice, and such a diet may not provide significant health benefits on its own, it is conceivable that integrating RES with a healthy diet, such as the Mediterranean diet, could offer concrete health advantages.

## 4 Conclusion

Chronic inflammation forms a positive feedback loop with oxidation, reinforcing each other and becoming part of the pathogenic mechanism proper to various chronic conditions, including PDS, CVD, type II DM, and RA. An effective therapeutic approach to interrupt this self‐sustaining mechanism is to switch off the inflammation by targeting directly its exogenous cause (e.g., the oral microbiota/ecosystem challenge) or/and addressing the different key points that drive acute inflammation to become chronic, thereby also reducing oxidative stress.

NSPT can effectively reduce chronic gingival and systemic inflammation in PDS patients after a short initial increasing phase.

The oxidative stress associated with the oral microbiota is also significantly reduced by NSPT. Omega‐3 PUFAs, CURs, and RES show favorable therapeutic results in patients with PDS, type II DM, CVS, and RA. Furthermore, PUFAs, CURs, and RES, in addition to NSPT, seem to provide significantly better clinical improvement.

Interestingly, periodontal therapy should be performed not only during the acute onset or relapse of PDS but also continuously as periodontal supportive therapy. It is prominent to deem dietary composition all the same, considering diet as a lasting health behavior that should be integrated into a biopsychosocial framework.

## Author Contributions

**Roberta Salvatori:** conceptualization (equal), data curation (equal), formal analysis (equal), investigation (equal), methodology (equal), supervision (equal), validation (equal), writing – review and editing (equal). **Luigi Generali:** investigation (supporting), methodology (supporting). **Elisa Bellei:** investigation (supporting), methodology (supporting). **Stefania Bergamini:** investigation (supporting), methodology (supporting). **Carlo Bertoldi:** conceptualization (equal), data curation (equal), formal analysis (equal), investigation (equal), methodology (equal), project administration (equal), supervision (equal), validation (equal), writing – original draft (equal), writing – review and editing (equal).

## Ethics Statement

The authors have nothing to report.

## Conflicts of Interest

The authors declare no conflicts of interest.

## Data Availability

Research data are not shared.
